# User-dependence of myocardial infarct identification using semi-automated thresholding techniques: implications for CRT response predictions based on scar burden

**DOI:** 10.1186/1532-429X-13-S1-P263

**Published:** 2011-02-02

**Authors:** Jonathan Suever, Matthew Janick, Stamatios Lerakis, John Oshinski

**Affiliations:** 1 Georgia Institute of Technology/Emory University, Atlanta, GA, USA; 2 Department of Cardiology, Emory University School of Medicine, Atlanta, GA, USA; 3 Department of Radiology, Emory University School of Medicine, Atlanta, GA, USA

## Objective

To assess the effect of inter-observer variability on myocardial infarct identification and quantification for the prediction of response to CRT.

## Background

Semi-automatic segmentation of infarcted tissue in late gadolinium-enhanced (LGE) MR images is usually done using a thresholding technique. In the threshold technique, an observer identifies a region of remote “normal” myocardium. Based on the distribution of pixel intensities within this “normal” region, pixels that lay more than two standard deviations outside the mean of this distribution are classified as infarcted myocardium.

Having a left ventricular scar burden greater than 33% has previously been used as a cut-off for predicting non-response to cardiac resynchronization therapy (CRT) (Chalil et al. Europace, 2007).

We ***hypothesized*** that the user-defined region of “normal” myocardium would have a significant effect on the classification of patients as non-responders to CRT (>33% LV scar burden).

## Methods

On a stack of short-axis LGE images from 22 patients with myocardial scar tissue present, two experienced observers manually traced: 1) endocardial and epicardial borders, and 2) a region of nulled (normal) myocardium. For each user, the mean and standard deviation of pixel intensities within the “normal” ROI were computed. Any pixel within the myocardium at least two standard deviations above this mean was classified as infarct. Using these values, scar burden estimates (% LV mass) were compared.

The two observers’ classification of each patient as a responder or non-responder was compared using Cohen’s kappa (κ), a measure of agreement of categorical measures.

## Results

When applying the 33% scar burden cutoff to the automatically segmented scar, the predicted response rates were 33% for observer 1 and 73% for observer 2. The observers agreed on the selection of non-responders only 60% of the time (κ=0.3) indicating poor agreement.

The discrepancy in response prediction can be partly attributed to the low correlation between the two observers using the automated method. A large inter-observer variation makes it difficult to reliably use a threshold for CRT response predication that is obtained using threshold methods for scar identification.

## Conclusion

Scar estimates relying upon threshold-based identification techniques are highly operator dependent. To use LGE scar burden values to select patients for CRT, a manual infarct border should be used, or more reliable infarct identification techniques must be developed for clinical use.

